# Increased resting state connectivity between ipsilesional motor cortex and contralesional premotor cortex after transcranial direct current stimulation with physical therapy

**DOI:** 10.1038/srep23271

**Published:** 2016-03-16

**Authors:** Joyce L Chen, Gottfried Schlaug

**Affiliations:** 1 Canadian Partnership for Stroke Recovery, Sunnybrook Research Institute 2075 Bayview Avenue, M6-176 Toronto, Ontario, M4N 3M5, Canada; 2 Department of Physical Therapy and Rehabilitation Sciences Institute University of Toronto Toronto, Ontario, Canada; 3 Department of Neurology; Neuroimaging and Stroke Recovery Laboratory Beth Israel Deaconess Medical Center and Harvard Medical School Boston, MA, USA

## Abstract

Non-invasive stimulation of the brain using transcranial direct current stimulation (tDCS) during motor rehabilitation can improve the recovery of movements in individuals with stroke. However, the neural substrates that underlie the clinical improvements are not well understood. In this proof-of-principle open-label pilot study, five individuals with stroke received 10 sessions of tDCS while undergoing usual care physical/occupational therapy for the arm and hand. Motor impairment as indexed by the Upper Extremity Fugl Meyer assessment was significantly reduced after the intervention. Resting state fMRI connectivity increased between ipsilesional motor cortex and contralesional premotor cortex after the intervention. These findings provide preliminary evidence that the neural underpinnings of tDCS coupled with rehabilitation exercises, may be mediated by interactions between motor and premotor cortex. The latter, of which has been shown to play an important role in the recovery of movements post-stroke. Our data suggest premotor cortex could be tested as a target region for non-invasive brain-stimulation to enhance connectivity between regions that might be beneficial for stroke motor recovery.

After a stroke, many individuals are left with motor impairment and dysfunction, which may impede independent living for months to even years^1^,^2^,^3^,^4^. Rehabilitation interventions currently applied typically involve exercises for the affected limb, geared towards re-learning of lost or impaired skills. There has been a growing interest in supplementing traditional therapy to further enhance motor stroke recovery, especially in those individuals with chronic stroke who may have the capability to improve but for whom there are limited therapeutic options^5^. These supplemental therapies may include enhancing sensory feedback^6^ and using robotic devices to increase exercise intensity and repetition^7^. Recently, there has been much interest in the simultaneous pairing of non-invasive brain stimulation, such as transcranial direct current stimulation (tDCS), with peripheral sensorimotor activities^8^,^9^,^10^. By electrically stimulating the brain, tDCS is thought to directly modulate and enhance neuroplasticity mechanisms if applied with peripheral sensorimotor activities^11^,^12^. Thus, when tDCS is applied to motor brain regions and is coupled with rehabilitation exercises, it has been shown to improve motor recovery more than if rehabilitation exercises were performed alone^13^,^14^,^15^,^16^,^17^,^18^. Given its clinical promise, there is a need to better understand the neural underpinnings for how tDCS coupled with rehabilitation may modulate neuroplasticity mechanisms of stroke recovery. Therefore, the objective of this proof-of-principle pilot study is to investigate the changes in neural connectivity that result after a 10 day intervention (over a two-week period) comprising tDCS coupled with physical and occupational therapy (PT/OT), in five chronic stroke patients.

Specifically, we use resting state functional magnetic resonance imaging (rsfMRI) to examine how the temporal coupling in neural activity (i.e. connectivity) between brain regions may change as a function of the intervention. An advantage of rsfMRI is that participants are scanned while “resting”, which thus avoids the problem of controlling for effort and performance when stroke patients with varying motor deficits are studied using the task-based fMRI approach^19^,^20^. To our knowledge, no studies have examined resting state connectivity changes associated with a combined tDCS + PT/OT intervention for stroke patients. In stroke patients, resting state interhemispheric connectivity between motor regions positively correlates with motor outcome; individuals with higher connectivity are less impaired and have better function^21^,^22^,^23^. Therefore, we explore whether resting state connectivity between interhemispheric motor regions would increase post-intervention.

## Results

### Behaviour

**Figure 1:**
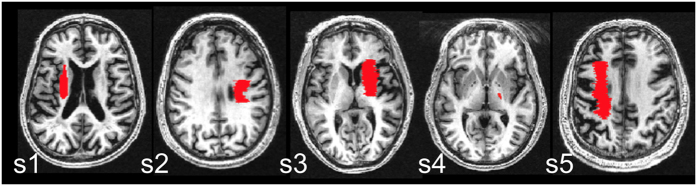
Lesion location. Lesions are shown overlaid on each subject’s T1-weighted image. The slice with the maximal lesion size is shown for each subject (S).

**Figure 2:**
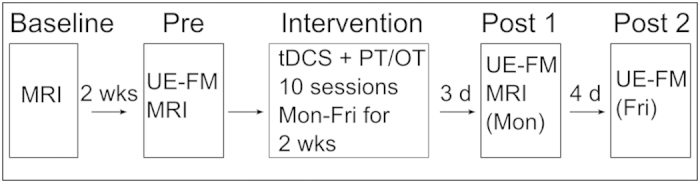
Study timeline. Magnetic resonance imaging: MRI; Upper Extremity Fugl-Meyer Assessment: UE-FM; Transcranial direct current stimulation: tDCS; Physical and Occupational Therapy: PT/OT; Monday: Mon; Friday: Fri; weeks: wks; days:d.

Five individuals with stroke (Fig. 1, Table 1) underwent 10 sessions (over two consecutive weeks, daily sessions from Monday-Friday) of bihemisphere tDCS paired simultaneously with PT/OT. Electrode placements were according to the model of impaired interhemispheric inhibition post-stroke^13^,^24^: the anode electrode was placed over the ipsilesional motor cortex (C3 or C4 according to the 10–20 EEG system) to up-regulate its neural activity, and the cathode electrode was placed over the contralesional motor cortex to down-regulate overactivity. Participants also underwent the Upper Extremity Fugl Meyer (UE-FM) assessment of motor impairment at three time points: before the intervention (pre), and at three (post 1) and seven (post 2) days after the intervention (Fig. 2). A paired samples t-test showed participants scored significantly higher on the UE-FM assessment at post 1 compared to pre intervention (t(4) = −6.41, p = 0.003, two-tailed). On average, participants improved 6.6 points (range 3 to 9 points) (Table 1). The effects at post 1 were maintained, as there were no significant differences in scores between post 1 and post 2 (t(4) = −1.45, p = 0.22, two-tailed).

## Mri

### Resting state connectivity of ipsilesional motor cortex

**Figure 3:**
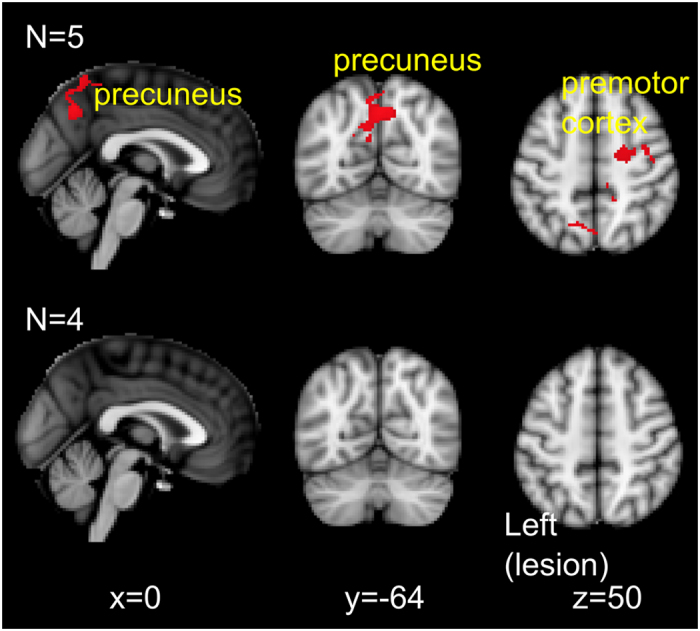
Resting state connectivity with left ipsilesional motor cortex. First row: regions with increased resting state connectivity with left motor cortex at post 1 relative to pre-intervention. Second row: no changes in resting state connectivity between baseline and pre-intervention time points. Results presented using cluster thresholding at z > 2.5, p < 0.05 corrected.

To address the specific aim of this pilot study, resting state functional MRI was obtained at two time points: before the start of the intervention (pre), and after the end of the intervention (post 1). Similar to prior work in stroke^21^,^22^,^23^, we used a seed-based connectivity approach to delineate voxels temporally correlated in neural activity with the ipsilesional and contralesional motor cortex. The primary analysis showed that after the intervention (post 1 relative to pre), there was increased resting state connectivity between the ipsilesional motor cortex seed region with ipsilesional and contralesional precuneus and contralesional premotor cortex (Table 2, Fig. 3: first row). In four of the five participants, another MRI scan was performed 2 weeks before the start of the intervention (baseline). This allowed us to examine any possible changes between two scans without an intervention in between (baseline vs pre) to ensure a stable baseline. This secondary analysis showed there were no changes in resting state connectivity between baseline and pre time points (Fig. 3: second row).

### Resting state connectivity of contralesional M1

There were no significant changes in connectivity between the contralesional motor cortex seed region with any voxels, for either the primary or secondary analyses.

## Discussion

In this proof-of-principle pilot study, individuals with chronic stroke underwent resting state fMRI before and after an intervention comprising 10 sessions of tDCS combined with PT/OT. Our findings demonstrate that at post-intervention, there was: 1) a significant reduction in motor impairment for all individuals, and 2) an increased resting state connectivity between ipsilesional motor cortex with contralesional premotor cortex and bilateral precuneus. These findings support prior literature on the use of bihemisphere tDCS as an adjuvant to rehabilitation exercises^13^,^17^,^25^, and provide preliminary evidence for the a possible neural substrate that underlie these behavioural changes.

Our findings corroborate prior research that show the potential of a tDCS + rehabilitation intervention in reducing motor impairment in chronic stroke patients^13^,^14^,^17^,^26^. Specifically, at 7 days after the last day of the intervention (post 2), participants showed between 5 to 15 points improvement on the UE-FM assessment of motor impairment compared to the pre-therapy assessment. These values are above the range reported (4.25–7.25 points) to represent a clinically important difference on this assessment in the chronic stroke population^27^. Thus, individuals in our pilot study demonstrated clinically meaningful change via reductions in motor impairment.

The reduction of motor impairment in the chronic stage of stroke may be difficult to achieve given the period of spontaneous or natural brain recovery has passed. Changes in motor ability in the chronic stage are thought to occur at the functional level where individuals can achieve a task goal with compensatory actions. Indeed, functional changes as measured by the Wolf Motor Function Test (WMFT)^13^ and Jebsen Taylor Hand Function Test (JTHFT)^17^,^28^,^29^ have been reported in the tDCS literature in chronic stroke. Interestingly, findings from two studies have shown changes in both assessments of impairment and function in the same patients^13^,^17^. In these studies, the intervention paired with tDCS was either standard physical/occupational therapy using a task-oriented approach^13^ or constraint induced movement therapy, which is also a task-oriented approach^17^. Thus, an intervention comprising tDCS with intense, task-oriented training may not only help chronic stroke patients improve function, but also reduce their motor impairment. This suggests that motor ‘recovery’ and ‘compensation’ mechanisms^30^ may both occur to enhance movements in the chronic stage post-stroke. Further studies are required to better understand how these mechanisms operate and interact in the chronic stage of stroke.

We also provide novel, though preliminary evidence that these clinically meaningful motor improvements may relate to increased resting state connectivity between ipsilesional motor cortex with contralesional premotor areas. In individuals with chronic stroke, those with less impairment have higher resting state connectivity between ipsilesional and contralesional motor brain regions^21^,^22^,^31^. Our findings additionally suggest that following an intervention comprising tDCS with PT/OT, resting state connectivity increased between ipsilesional motor cortex and contralesional premotor cortex; no changes were detected during a similar period of no intervention, which was done prior to the intervention period for 4 of the 5 subjects. This finding supports the notion that interactions between ipsilesional motor cortex and contralesional premotor cortex may have an important role in the post-stroke recovery of function^32^, especially in individuals with more clinical impairment^33^,^34^. Specifically, neural activity in contralesional premotor cortex is thought to contribute to motor performance^35^, perhaps facilitating ipsilesional motor activity^36^, in individuals with chronic stroke.

The role of the ipsilesional PMd may also be important for individuals with good motor recovery^37^. In particular, it has been suggested that ipsilesional PMd may be implicated in the recovery of individuals with small lesions to the motor cortex and/or corticospinal tract^33^. However, we did not find significant changes in resting state connectivity between this region and ipsilesional motor cortex. On reason for this may be that in our small sample, participants had a range of motor impairment (FM-UE scores from 21 to 48) that would not be classified as well recovered. It is thought that contralesional PMd is implicated in the recovery individuals with greater damage to motor cortex and/or corticospinal tract, and thus who are not well recovered^33^.

Lastly, we did not find any significant changes in resting state connectivity using the contralesional motor cortex seed ROI. A potential reason for this may be that in the individuals tested, the mechanism of recovery was mediated by ipsilesional motor cortex with its interactions via resting state connectivity to other regions.

Our findings unexpectedly showed increased resting state connectivity between ipsilesional motor cortex with bilateral precuneus. In healthy individuals, the dorsal-anterior portion of the precuneus demonstrates resting state connectivity with primary motor cortex^38^. Studies have attributed the role of the precuneus in multisensory integration^39^ and visuospatial processing^40^, which may also entail motor imagery^41^. During the course of the intervention, participants may have engaged these processes in relation to the execution of upper extremity movements. However, given connectivity of ipsilesional motor cortex was with bilateral precuneus, it is nonetheless unclear how these findings may directly relate to the arm/hand used during the intervention. Future work could explicitly test whether multisensory integration or visuospatial processes for example, are indeed improved post-intervention, and whether they relate to an increased precuneus-motor connectivity.

Some limitations of this work include the fact that we have a small sample size. However, given the aim of this work is proof-of-principle, we have now demonstrated the scientific potential for using resting state fMRI to study mechanisms related to improvements from a tDCS with PT/OT intervention in a larger sample. As well, our experimental design included a baseline period of no intervention where no changes in resting state connectivity occurred. However, future work will incorporate a separate control group of individuals with chronic stroke who undergo PT/OT with sham tDCS. This would allow us to further dissect the effects seen in this pilot study and determine what proportion can be attributed solely to the effects of tDCS.

The identification of nodal points within a critical network of brain regions involved in the recovery process might represent novel targets of brain-stimulation to enhance stroke recovery. Our network analysis showed resting-state connectivity changes that highlight the potential role of premotor cortex in facilitating recovery, which could potentially be further enhanced by non-invasive brain-stimulation.

## Methods

### Participants

Five participants (2 female; mean age 57.4 ± 12.9 years, mean time since stroke 11.4 ± 6.5 months; all right-handed) gave written informed consent to participate in this pilot study that was approved by the Institutional Review Board of Beth Israel Deaconess Medical Center, Boston, USA. All methods were carried out in accordance with approved institutional guidelines and regulations. Inclusion criteria were as follows: first ischemic stroke in the middle cerebral artery territory, greater than 3 months since stroke onset, no previous or subsequent cerebral ischemia or haemorrhage, and no history of seizures. In addition, to be eligible for the tDCS intervention, participants were required to show presence of movements in the wrist (at least 10 degrees dorsiflexion) and/or fingers (at least 10 degrees of flexion and extension). Exclusion criteria were as follows: severe deficits in cognition, comprehension and clinically apparent neglect that would preclude meaningful participation in the intervention. The co-author GS is a stroke neurologist and the lead-author JLC a trained physical therapist. We used clinical judgment and routine neurological exams during the recruitment interview/assessment to evaluate these exclusion criteria.

### Intervention

The intervention involved an experienced occupational therapist using usual care/standard PT/OT techniques (60 minutes) to treat the arm and hand, while bihemisphere tDCS was concurrently applied for the first 30 minutes. All participants received similar exercises that comprised of task-oriented functional training, which promoted sensorimotor integration and the coordination of movement. The intervention was catered to the capabilities of the participants. Our prior work implemented a sham-controlled randomized trial and showed reduced motor impairment and improved motor function in the real tDCS stimulation group^13^,^14^. Since these previous trials showed efficacy over a sham intervention, the current study was conducted as an open label study. The aim of the present open-label proof-of-principle pilot study is to understand how resting state fMRI connectivity may be changed by the tDCS + PT/OT intervention. No blinding was implemented in this open-label study as every participant received the intervention.

tDCS was delivered using a Phoresor II DC autostimulator (IOMED, Salt Lake City, UT) through 2 saline-soaked surface gel-sponge electrodes (16.3 cm^2^) for 30 minutes at 1.5 mA direct current. The anode is placed either at the C3 or C4 landmark of the international 10–20 EEG system, depending on which hemisphere had the ischemic stroke. The cathode electrode is placed over contralesional motor cortex (either C3 or C4). Together, these electrode and stimulation parameters have been applied in prior work that has shown the combination of tDCS and PT/OT to reduce motor impairment and improve function^13^.

### Assessment

Each participant underwent the UE-FM assessment^42^, which consists of 30 voluntary UE motions observed by a rater and 3 tendon tap responses. Ordinal ratings (2 = near normal ability/response, 1 = partial ability, 0 = unable to perform or no response) for each item are summed and reported out of 66 points with a lower score representing more motor impairment.

### MRI acquisition

Images were acquired on a 3T GE scanner. Resting state fMRI was performed with T2* weighted EPI slices acquired every 2 seconds (TR = 2 s, TE = 25 ms) for a total of 300 volumes. We obtained 28 horizontal slices covering the whole brain with in an-plane resolution of 3.75 × 3.75 mm^2^, slice thickness of 4 mm, 1 mm gap (matrix size = 64 × 64 mm^2^, field of view = 240 × 240 mm^2^). Subjects fixated their eyes on a red cross during volume acquisition (~10 minutes). A pulse oximeter was placed on the participants’ unaffected index finger to record heart rate, and a pneumatic belt was placed around the chest to record respiration. These data were sampled at 40 Hz.

High-resolution T1-weighted structural images were also acquired (resolution = 0.9375 × 0.9375 × 1.5 mm^3^, matrix size = 256 × 256 mm^2^, field of view = 240 × 240 mm^2^, TE = 2.8 mx, TR = 6.6 ms, flip angle = 15 degrees), along with a set of axial fluid-attenuated inversion recovery (FLAIR) images (0.5 × 0.5 × 0.5 mm^3^) to assist with lesion size determination. As per methods from our prior work^43^,^44^, we also determined the corticospinal tract (CST) lesion load, which quantifies the degree to which the stroke lesion overlaps a canonical CST (Table 1).

### MRI Analysis

All data were analysed using FSL tools (http://www.fmrib.ox.ac.uk/fsl)^45^. Prior to any analysis, images for three participants who had right hemisphere lesions were flipped along the x-plane so that all lesions are on the left hemisphere (thus, left = ipsilesional hemisphere).

#### Registration

Resting state fMRI data were registered to standard space using *FNIRT* (FMRIB’s Non-linear Registration Tool) with an affine transformation. Lesions were drawn in each subject’s structural space on the T1-weighted images using the co-registered FLAIR image as a reference (Fig. 1). The lesion mask was used during registration steps of the structural and resting state fMRI data such that voxels in lesioned regions were excluded from the normalization procedure.

#### Region of interest

We used a seed-based connectivity approach to delineate voxels temporally correlated in neural activity with the ipsilesional and contralesional motor cortex. Therefore, region of interest (ROI) masks in ipsilesional and contralesional motor cortex were created in standard space using a combination of the precentral gyrus mask from the Harvard-Oxford atlas and the Jülich BA4a and BA4p masks, available from FSL. The Harvard-Oxford precentral mask was first thresholded by 20%, and binned. Voxels inferior to z = 62 were clearly segregated to either the precentral gyrus or SMA; we excluded voxels in the SMA. For voxels superior to z = 62, the SMA was defined as encompassing five voxels in the +/−x direction from the midline; those voxels greater than +/−five voxels from the midline were considered to be part of the precentral gyrus. The mask was overlaid on the MNI152 2 mm standard brain and voxels that were posterior to the central sulcus were also excluded. The Jülich BA4a and BA4p masks were thresholded at 10% and the anterior boundary was used to segregate the precentral gyrus into the posterior bank of the precentral gyrus (BA4a and BA4p) and premotor cortex (remaining, mostly anterior bank of precentral gyrus). The resulting masks were non-linearly transformed into the resting state fMRI space of each subject, using *FNIRT*.

#### Resting state fMRI analysis: single-subject level

For each participant, we used *FEAT* (FMRIB’s Expert Analysis Tool) to pre-process the data (described below), and modeled nuisance regressors of no-interest that include cerebrospinal fluid (CSF), white matter (WM), head motion, and physiological noise (described below). Thus, the residuals from this analysis have all nuisance regressors removed. We then calculated the mean time course of the blood-oxygenation level dependent signal (BOLD) in all voxels of the ipsilesional and contralesional motor cortex ROI, separately, from the residuals. The mean motor cortex time series were then entered separately, as an explanatory variable (EV) in the general linear model where we determined, for each participant, voxels where BOLD was temporally correlated with that of ipsilesional and contralesional motor cortex. Thus, this whole-brain analysis allows us to extract the so-called motor network, identifying regions whose BOLD signal is temporally correlated with the seed motor cortex^46^.

#### Pre-processing

We used *FEAT* to pre-process the data for each participant. The first six volumes were discarded to account for T1-saturation effects and to achieve steady state of the spin system. The images were then motion corrected by realignment to the middle volume of each run, spatially smoothed with a Gaussian kernel of 8 mm full-width at half maximum, high-pass temporal filtered at 0.01 Hz, and grand mean intensity normalized. *BET* (Brain Extraction Tool) was used to remove signal from non-brain tissue.

#### Nuisance variables

The time series for nuisance variables were computed and used as regressors of no-interest. These included cerebrospinal fluid (CSF), white matter (WM), head motion, and physiological noise. *FAST* (FMIRB’s automated segmentation tool) was used to segment each individual’s structural image. The resulting CSF and WM images were then eroded to ensure only voxels in the CSF and WM were included. These masks were transformed into the resting state fMRI space using *FLIRT* (FMRIB’s linear registration tool) with 6 degrees of freedom. The mean time courses of the CSF and WM signal were extracted from all voxels within the respective masks in the preprocessed data. Six motion parameters (x, y, and z translations and rotations) derived from motion correction were also obtained for each individual. Lastly, the PhLEM Toolbox (http://sites.google.com/site/phlemtoolbox/)^47^ was used to generate regressors for the physiological data. We modeled the respiration and cardiac cycles, respiration volume and heart rate, using the *Retroicor* and *Variation* models in PhLEM.

#### Resting state fMRI analyses: group level

Two group-level analyses were performed. The primary analysis included all five participants where we performed a paired t-test to compare connectivity of the ipsilesional motor cortex immediately before (pre) and after (post 1) the 10-day intervention. The same procedure was also performed for contralesional motor cortex. Thus, these analyses determine whether there are differences in connectivity patterns before and after the intervention, within the motor network identified from the single-subject level analysis described above. A second analysis was performed to compare the two scan points obtained before the start of the intervention in four individuals (baseline vs pre). Here, we expect no change in connectivity between time points where no intervention occurred. Results are considered significant if they meet a cluster threshold of z > 2.5, p < 0.05 corrected.

## Additional Information

**How to cite this article**: Chen, J. L. and Schlaug, G. Increased resting state connectivity between ipsilesional motor cortex and contralesional premotor cortex after transcranial direct current stimulation with physical therapy. *Sci. Rep.* **6**, 23271; doi: 10.1038/srep23271 (2016).

## Figures and Tables

**Table 1:** Patient Characteristics.

Subject ID	Age at assessment (years)	Hemisphere stroke	Sex	Time since stroke (months)	Lesion Size (cm^3^)/wCST-LL (cc)	Upper Extremity Fugl Meyer Assessment		
						Pre	Post 1	Post 2
1	77	Left	M	4	3.98/2.25	23	26	28
2	49	Right	F	16	7.02/2.21	41	47	51
3	50	Right	F	9	11.86/1.86	32	40	39
4	47	Right	M	8	0.87/2.35	48	55	54
5	64	Left	M	20	9.46/4.37	21	30	36

wCST-LL: weighted corticospinal tract lesion load (the degree to which the stroke lesion overlaps with the corticospinal tract^43^).

**Table 2:** Regions of significantly increased resting state connectivity with left motor cortex at post 1 compared to pre intervention.

Cluster	Number of voxels in cluster	Z-score (local maxima)	MNI Coordinates		
			X	Y	Z
Precuneus	676	3.42	4	−58	66
		3.31	0	−62	44
		3.30	8	−66	42
		3.25	−12	−62	32
		3.18	10	−32	54
		3.18	−20	−76	34
Premotor cortex	397	3.16	32	−6	32
		3.15	26	−6	44
		3.12	38	−12	30
		3.06	20	−10	48
		3.02	40	−12	48
		2.98	34	−6	46
