# Erratum: Increased resting state connectivity between ipsilesional motor cortex and contralesional premotor cortex after transcranial direct current stimulation with physical therapy

**DOI:** 10.1038/srep24960

**Published:** 2016-04-29

**Authors:** Joyce L. Chen, Gottfried Schlaug

Scientific Reports 6: Article number: 23271; 10.1038/srep23271 published online: 04162016; updated: 04292016.

In the HTML version of this Article, Gottfried Schlaug is incorrectly listed as being affiliated with ‘Department of Physical Therapy and Rehabilitation Sciences Institute University of Toronto Toronto, Ontario, Canada’. The correct affiliation is listed below:

Department of Neurology; Neuroimaging and Stroke Recovery Laboratory Beth Israel Deaconess Medical Center and Harvard Medical School Boston, MA, USA.

